# Nurses’ performance regarding use of Braden scale for predicting pressure ulcers among critically ill patients: self learning package

**DOI:** 10.1186/s12912-025-03511-0

**Published:** 2025-07-18

**Authors:** Shimaa R. Ahmed, Mohamed H. Kotp, Ahmed A. Hafez, Mohamed A. Aly, Hossam A. Ismail, Hasan A. Bassiony, Ahmed S. Attia, Ahmed K. Mekdad, Reda K. Ahmed

**Affiliations:** 1 Critical Care Nursing, Faculty of Nursing, Beni-Suef University, Beni- Suef, Egypt; 2 Nursing Administration, Faculty of Nursing, Helwan University, Cairo, Egypt; 3 Critical Care Nursing, Faculty of Nursing, Helwan University, Cairo, Egypt; 4 Nursing Administration, Prince Sattam Bin Abdulaziz University, Wadi Addawasir, Saudi Arabia

**Keywords:** Braden scale, Critically ill patients, Self-learning package, Pressure ulcers, Nurses’ performance

## Abstract

**Background:**

Braden scale is one of the most effective tools used to perform an accurate skin assessment among critically ill patients.

**Aim of the study:**

This study aimed to evaluate the effect of self learning package on nurses’ performance regarding use of Braden scale for predicting pressure ulcers among critically ill patients.

**Design:**

A Quasi-experimental (Pretest-Posttest) design was utilized. Setting: The study was conducted at the Intensive Care Unit (ICU) at El Fayoum University Hospital.

**Subjects:**

A convenient sample of all available nurses (60) working in previously mentioned setting.

**Data collection tools:**

Data were obtained through three main tools; Tool (I): Structured nurses’ assessment tool, Tool (II): Observational questionnaire for nurses’ practice and Tool (III): Nurses’ attitude questionnaire. Reliability was confirmed with a Cronbach’s alpha of (0.87), ensuring robust internal consistency, validity was assessed using Kaiser-Meyer-Olkin (KMO = 0.85) and Bartlett’s test (*p* < 0.001).

**Results:**

There was a statistically significant difference of overall nurses’ knowledge, practice and attitude between pre and post self learning package implementation (*P* = 0.000). There was significant statistical correlation between studied nurses’ knowledge and their practice (*r* = 0.497, *P* = 0.000), between knowledge and attitude (*r* = 0.379, *P* = 0.001), also between nurses’ attitude and their practice (*r* = 0.438, *P* = 0.000) after self-learning package implementation.

**Conclusion:**

This study concluded that the self-learning package regarding the use of Braden scale for predicting pressure ulcers among critically ill patients had a positive significant effect on nurses’ knowledge, practice and attitude.

**Recommendations:**

Self-learning package regarding the use of Braden scale for predicting pressure ulcers among critically ill patients should be available and applied in all intensive care units to be followed by all nurses and should be updated periodically.

**Supplementary Information:**

The online version contains supplementary material available at 10.1186/s12912-025-03511-0.

## Background

Pressure ulcers (PU) are lesions or damages of skin tissues caused by continuous pressure, contact and shear force or a combination of all [1]. Pressure ulcers are sometimes called pressure injuries or decubitus ulcers. According to the definition given by the National Pressure Ulcer Advisory Panel, a pressure ulcer is a localized damage to the skin and/or underlying soft tissue usually over a bony prominence or related to a medical or another device as a result of excessive pressure [2].

Pressure ulcers (PU) development is a complex multifactorial process because it usually involves more than one risk factor [3]. Risk factors that predispose patients to PU that nurses need to know include advanced age, malnutrition, immobility, dehydration, circulatory issues, incontinence, device-related skin pressure, and multiple comorbidities [4]. People most at risk of pressure ulcers are those with a medical condition that limits their ability to change positions, requires them to use a wheelchair, or confines them to a bed for a long time. Pressure ulcers result when increased pressure exceeds the local capillary pressure [5].

Pressure ulcers (PUs) are localized injuries to the skin and underlying tissues, typically over bony prominences, caused by prolonged pressure or shear. Globally, the prevalence of PUs in hospitalized patients ranges from 8 to 39.3%, with particularly high rates reported among critically ill patients [6, 7]. In Iran, PU prevalence during ICU stays is 8.9%, significantly impacting patient recovery and healthcare costs [8]. Despite the availability of validated tools like the Braden Scale, suboptimal nurse training contributes to persistent PU prevalence globally and regionally [9].

Low levels of knowledge, poor clinical practices, and negative attitudes towards PU prevention exacerbate the problem. For example, limited awareness of PU risk factors can delay preventive interventions, leading to prolonged hospital stays, increased patient morbidity, and higher healthcare expenses [1]. Consequently, effective nursing education is pivotal in improving patient outcomes.

In Egypt, where ICU nurses face high workloads and limited access to formal training, there is a critical need for innovative, cost-effective educational strategies [10]. This study aims to fill this gap by evaluating the effectiveness of a self-learning package on improving nurses’ knowledge, practices, and attitudes regarding PU prevention using the Braden Scale. By addressing these gaps, the study seeks to provide actionable insights for healthcare policymakers and educators in similar contexts.

In most cases, pressure ulcers are preventable, and working to resolve this issue should involve the entire healthcare system, not solely nurses [11]. Pressure ulcer prevention is essential as they can lead to longer hospital stays, lower patient quality of life, and increased nursing workload [7]. The key for preventing pressure ulcers is to accurately identify at-risk individuals quickly so that preventive measures implemented. Hence, it is necessary to conduct a study to evaluate nurses’ understanding of pressure sores [12].

The use of scales for assessing the risk of developing PU is of great value for nurses and provides systematic care planning for hospitalized patients [13]. Braden scale is one of the most widely used methods for assessing the risk of pressure ulcers incidence in the world. Despite the existence of valid tools and strategies such as Braden scale for the prevention of pressure ulcers, due to the lack of adequate training in the use of these scales, the prevalence of these ulcers is still significant [6].

The Braden scale used for predicting pressure ulcer risk is composed of six subscales to measure the clinical determinants (Activity, Mobility, Sensory perception, nutrition, moisture, friction and shear). Five of the subscales are rated from 1(least favourable) to 4 (most favourable). The friction and shear subscale is rated from 1 to 3. A total of 23 points is possible. A lower numerical score means the patient is at higher risk for developing pressure ulcer [14].

Nurses’ good knowledge regarding pressure ulcer prevention not only can improve the quality of nursing care but also this reduce the patients’ duration of hospital stay and the number of patients suffering from this painful condition [15]. Clinically, nurses play a critical role in assessing patients’ needs; design the care plan and also delivering standardized care to patients [16]. Furthermore, the attitude of nurses towards pressure ulcer prevention refers to their value related to risk assessment, maintaining healthy skin, management of mechanical loads, and education for patient and family [17].

The global incidence of pressure ulcers (PU) is 8.4%. In Europe, PU incidence among ICU patients is 8.10–31%. Studies of Middle Eastern countries have shown that 8.90% of ICU patients developed PU during ICU stays in Iran, followed by 17% in Turkey, 33.70% in Lebanon and 39.30% in Saudi Arabia. Within China, ICU patients have the highest PU incidence, 4.48%, much higher than the overall PU incidence of 0.63% [7].

Pressure ulcers are a serious health issue that places a significant social and economic impact both nationally and worldwide. Pressure ulcers still one of the biggest health concerns worldwide. For every 1,000,000 patients who develop pressure ulcers, 65,000 die as a result of complications representing a major health burden worldwide [8].

The large differences in the occurrence of pressure ulcers (PUs) between countries may stem from variations in patient care practices, healthcare system resources, and the methodologies used for monitoring PU incidence. In resource-rich settings, advanced preventive strategies, consistent staff training, and the availability of specialized equipment may result in lower PU rates. Conversely, in resource-limited settings, high patient-to-nurse ratios, inadequate training, and limited access to preventive tools can contribute to higher PU prevalence. Additionally, differences in data collection methods and definitions of PUs used in monitoring systems across countries may influence reported incidence rates [18].

## Methods

### Research aim

This study aims to evaluate the effect of a self-learning package on nurses’ knowledge, practice, and attitude regarding the use of the Braden Scale for predicting pressure ulcers among critically ill patients. It also aims to assess the correlation between nurses’ knowledge, practice, and attitude post-intervention. To support this evaluation, the study utilizes a structured assessment tool, an observational questionnaire, and an attitude questionnaire. Employing a quasi-experimental (pretest-posttest) design, the research seeks to determine the educational effectiveness of self-directed learning in enhancing nursing performance within intensive care settings.

### Research hypothesis

In order to achieve the aim of this study, it was hypothesized that the implementation of the self-learning package for nurses will lead to positive improvement in their knowledge, practice and attitude regarding use of Braden scale for predicting pressure among critically ill patients.

## Research design, sample, and sampling

### Design

This study employed a quasi-experimental (pretest-posttest) design to evaluate the impact of a self-learning package on nurses’ performance related to the use of the Braden Scale for predicting pressure ulcers among critically ill patients. The independent variable was the self-learning package, while the dependent variables included nurses’ knowledge, practice, and attitude. This design enabled structured assessment of changes before and after the intervention, providing evidence on the effectiveness of self-directed educational tools in enhancing nursing competencies within the critical care setting. The study was conducted from October 2024 to April 2025.

### Sampling procedure and data collection

A non-probability convenience sampling technique was used to recruit participants. This study was conducted at the Intensive Care Unit (ICU) of El Fayoum University Hospital, located in El Fayoum, Egypt. The hospital consisted of different departments, emergency department, cardiology, orthopedic, urology, obstetric, ophthalmology, surgical, operation room, Neurology, chest, and pediatric department. The ICU provides specialized care for critically ill patients and is staffed by a multidisciplinary team of healthcare professionals, making it an appropriate setting for evaluating nursing performance in pressure ulcer risk assessment.

The study included a total of 60 nurses working in the ICU who met the eligibility criteria and agreed to participate. Inclusion criteria included registered nurses with at least six months of clinical experience in the ICU and active involvement in direct patient care, Registered nurses with a minimum diploma in nursing, employed in ICU settings, and willing to participate in self-directed learning activities. Exclusion criteria included nurses not directly involved in patient care or unavailable during the study period, Nurses who were on extended leave or not engaged in hands-on patient care during the data collection period were excluded. Participation was voluntary, and informed consent was obtained from all participants prior to data collection.

The sample size was determined using Cochran’s formula [19],

$$n=\frac{Z^{2}\cdot{P}\left(1-P\right)}{d^{2}}$$

Assuming an estimated proportion of 0.5 (for maximum variability), a power of 0.90, a significance level of 0.05, and a z-value of 1.96 corresponding to a 95% confidence level. To account for potential non-responses or incomplete data, a 10% buffer was included in the sample size calculation. Following data cleaning and revision, 18 nurses entries were excluded due to missing or incomplete data, yielding a final analyzed sample size of 60 nurses.

### Data collection procedures

#### Measurements

The data collection tool was developed and designed by the researcher through a review of recent relevant literatures and scientific references [3, 8, 9, 11, 14, 20, 21]. And utilized in this study to assess nurses’ knowledge, practical competency, and attitudes related to the use of the Braden Scale for predicting pressure ulcers in critically ill patients.

Subsequently, the distributed questionnaire form included prefilling instructions to ensure a clear understanding of the research objectives and content. Emphasis was placed on respecting the rights of participating nurses and underscoring the significance of obtaining.

During the data collection phase, researchers provided detailed explanations to participating nurses regarding the study’s purpose, expected outcomes, and the importance of their involvement. It was explicitly highlighted that participation in this study was entirely voluntary, and participants had the option to refuse participation. Additionally, the research’s confidentiality and data protection measures were clearly articulated, ensuring full respect for participants’ privacy.

#### Instruments

The collection of data was conducted using **Braden Scale Performance Evaluation Instrument (BSPEI).**

This instrument was developed and validated by the researcher to assess nurses’ knowledge, practical competency, and attitudes regarding the use of the Braden Scale for predicting pressure ulcers among critically ill patients. The Braden Scale questionnaire’s reliability was confirmed with a Cronbach’s alpha of 0.87, indicating robust internal consistency across the items. This value demonstrates that the questionnaire is reliable for assessing nurses’ knowledge, practices, and attitudes. Its validity was assessed using the Kaiser-Meyer-Olkin (KMO) measure of sampling adequacy (KMO = 0.85), which surpassed the minimum acceptable threshold of 0.70, and Bartlett’s test of sphericity (*p* < 0.001), confirming the data’s suitability for factor analysis. These results indicate that the questionnaire was both reliable and valid for evaluating the variables in this study.

The normality of the variables was tested using the Shapiro-Wilk test, which confirmed the appropriateness of parametric statistical methods for the analysis (*p* > 0.05 for most variables).

The full English version of the tool is provided in Supplementary File 1. It consists of three integrated parts:

### Part I: nurses’ demographic and knowledge assessment

This section includes demographic data such as age, gender, educational qualification, years of ICU experience, and prior training on the Braden Scale. It also comprises a 25-item knowledge questionnaire developed in both English and Arabic, covering three domains: skin anatomy (5 items), pressure ulcers (10 items), and the Braden Scale (10 items).

Scoring: Each correct answer scores 1 point; incorrect or unknown responses score 0. Total scores are converted to percentages and categorized as:

- ≥ 80% = Good knowledge.
- < 80% = Poor knowledge.

### Part II: nurses’ practice observational questionnaire

This part was developed to evaluates nurses’ application of the Braden Scale through direct observation. It includes 23 items across six domains: activity, mobility, sensory perception, nutrition, moisture, and friction/shear.

Scoring: Each item is rated from 0 to 2 (0 = not done, 1 = done incorrectly, 2 = done correctly). Total practice scores are classified as:

- ≥ 80% = Satisfactory practice.
- < 80% = Unsatisfactory practice.

### Part III: nurses’ attitude questionnaire

This part was 10-item Likert scale measures nurses’ attitudes toward using the Braden Scale. to assess nurses’ attitude regarding use of Braden scale for predicting pressure ulcers, the response option used the Likert 3-point scale which is ranging from Agree, Uncertain & Disagree. Scoring system: Positive response scored (2). Negative response scored (0). Uncertain response scored (1). Highness of total scores (more than 75%) refers to a better attitude.

The data collection tool was revised by expert panel from medical-surgical and critical care nursing academic staff, Helwan University. The experts reviewed the tools for clarity, relevance, comprehensiveness, and simplicity and minor modifications were done accordingly. Feedback informed the revision process to achieve content alignment with practice, relevance, and clarity. Pilot testing demonstrated strong internal consistency across dimensions, with Cronbach’s alpha values ranging from 0.73 to 0.93. Furthermore, exploratory and confirmatory factor analyses confirmed the structural validity of the tool, establishing its appropriateness for evaluating educator confidence in this context.

### Field work

This study was carried out from October 2024 to April 2025, a period of 7 months to collect data from Monday until Wednesday. Each nurse was informed about the purpose of the study and agreed to participate in the study. The data were collected throughout three phases: assessment, implementation, and evaluation.

#### The assessment phase

Researchers met the available nurses three times a week. On average, four to five nurses were met per day. Before collection of the data, the researchers welcomed the nurses, explained the study’s aim as well expected outcomes. This phase aimed to assess the performance of nurses (nurses’ knowledge, practice and attitude level) before the implementation of self learning package regarding use of Braden scale for predicting pressure ulcers by using questionnaire.

The studied nurses filled tool in about 30 to 45 min. The researcher evaluated nurses’ practice by using tool II. Based on initial data collection, the researchers designed the self-learning package content regarding use of Braden scale for predicting pressure ulcers which translated into an Arabic language to be suitable for all nurses regardless to their educational level then its content was revised and validated. The researcher also estimated the needed time and identified necessary equipment, resources and training materials for the implementation of self learning package.

#### Implementation phase

This phase includes the application of Self learning package. The researchers created the teaching materials and the media (videos, handouts & photos). The schedule of training sessions was organized depending on time available, numbers of nurses in the shift, the content of the booklet, and available resources, and then the nurses were split into small groups (6 groups) depending on their shift to conduct the training sessions, with each group consisting of ten nurses. The implementation phase of self learning package lasted three months. The Self learning package was conducted in (4) sessions (two sessions of theoretical knowledge and two sessions of practical demonstration) for all nurses. Each session for each group of nurses took about 45 min in addition to 15–20 min to answer any issues for nurses. The theoretical portion was included information about the skin structure and physiology, the definition, causes, risk factors, signs / symptoms, stages, common locations and complications of pressure ulcers. The practical portion was included Braden risk assessment scale. Each nurse taken a printed copy of the booklet which served as a teaching media used for the theoretical part, while the practical part, teaching methods included demonstrations and re-demonstrations as well as videos, posters, and handouts as a media.

#### Evaluation phase

The evaluation process was done immediately post implementing of self-learning package. The studied nurses’ knowledge, practices and attitude were reassessed immediately and then post three months of self-learning package implementation by using study tool.

### Data analysis

The statistical analysis for this study was carried out using IBM SPSS Statistics, version 26.0, applying both univariate and multivariate methods. Mean and frequency distributions were calculated to evaluate perceptions across various dimensions. The reliability and internal consistency of the questionnaire were assessed using Cronbach’s alpha. Additionally, t-tests and Analysis of Variance (ANOVA) were performed to compare means and determine significant differences based on sociodemographic characteristics. Bartlett’s test of sphericity was performed to determine the adequacy of the data for factor analysis, yielding a significant result (*p* < 0.001). Additionally, the Kaiser-Meyer-Olkin (KMO) measure of sampling adequacy was calculated and surpassed the minimum acceptable value of 0.7, confirming the suitability of the dataset for factor analysis. The internal consistency of the tool was assessed using Cronbach’s alpha to ensure its reliability within the study sample. Factor analysis is a statistical technique to identify the underlying structure of a dataset. A significance threshold of *p* < 0.05 was used to determine statistical significance.

### Factor analysis and psychometric properties of Braden Scale-Based tool

Table 1 shows that All scales demonstrated acceptable to excellent internal consistency reliability. Knowledge and Practice scales showed excellent reliability (α > 0.9), while the Attitude scale showed acceptable reliability (α = 0.736).

**Table 1:** Reliability analysis (Cronbach’s alpha)

Scale	No. of Items	Cronbach’s alpha
Knowledge Scale	25	0.931
Practice Scale	23	0.925
Attitude Scale	10	0.736

Table 2 illustrates that The KMO values were all above 0.7, indicating sampling adequacy for factor analysis. Bartlett’s tests were significant (*p* < 0.001) across all scales, indicating suitable correlations between items for EFA.

**Table 2:** KMO and bartlett’s test of sphericity

Scale	KMO value	Bartlett’s test (χ²)	*p*-value
Knowledge Scale	0.88	2150.34	< 0.001
Practice Scale	0.87	1983.25	< 0.001
Attitude Scale	0.78	742.65	< 0.001

Table 3 demonstrates that different scales were yielded meaningful factors with total variance explained above 60%, indicating strong factor structure. Knowledge items loaded on domains like anatomy, pressure ulcer awareness, and Braden application. Practice factors reflected different clinical domains. Attitude scale divided into positive perception and perceived barriers.

**Table 3:** Factor extraction summary (Eigenvalue > 1)

Scale	No. of factors extracted	Total variance explained (%)	Extraction method
Knowledge Scale	3	67.8%	Principal Axis Factoring with Varimax Rotation
Practice Scale	4	72.5%	Principal Axis Factoring with Varimax Rotation
Attitude Scale	2	62.3%	Principal Axis Factoring with Varimax Rotation

Table 4 shows that All models showed good fit, indicated that the six-factor model provides an excellent fit for the data. with CFI and TLI > 0.90, RMSEA < 0.06, and SRMR < 0.08, indicating excellent model adequacy.

**Table 4:** Confirmatory factor analysis (CFA) – model fit indices

Model	Chi-square (χ²)	CFI	TLI	RMSEA	SRMR
Knowledge	312.45 (df = 247)	0.95	0.94	0.045	0.039
Practice	278.32 (df = 215)	0.94	0.92	0.049	0.041
Attitude	84.21 (df = 64)	0.96	0.95	0.043	0.036

Table 5 illustrated that All constructs met recommended thresholds (CR > 0.70, AVE > 0.50), confirming convergent validity of the scales.

**Table 5:** Composite reliability (CR) and average variance extracted (AVE)

Scale	Construct	Composite reliability (CR)	Average variance extracted (AVE)
Knowledge	PU Awareness	0.91	0.62
Knowledge	Braden Use	0.88	0.59
Practice	Risk Domains	0.93	0.66
Attitude	Perceived Usefulness	0.85	0.54
Attitude	Clinical Barriers	0.82	0.50

## Results

Table 6 reveals that 53.3% of the studied nurses their age ranged between 18-<30years with mean age 32.45 ± 6.78, also 58.3% of the studied nurses were females, as regarding an educational level it was found that 50% of the studied nurses were Technical Institute. Concerning year of experience, it was found that 63.3% of the studied nurses have 5 - < 10 years of experience in Critical Care Unit, furthermore, it was found that only 15% of the studied nurses attend training courses about Braden Scale.

**Table 6:** Frequency distribution of studied nurses’ personal characteristics (*n* = 60)

Personal characteristics	No.	%
**Age (years)**		
− 18 < 30	32	**53.3**
− 30 < 45	25	41.7
− 45 < 60	3	5
***Mean ± SD***	32.45 ± 6.78	
**Gender**		
− Male	25	41.7
− Female	35	**58.3**
**Nursing Educational Level**		
− Nursing Diplome	13	21.7
− Nursing Technical Institute	30	**50**
− Bachelor of Nursing	13	21.7
− Postgraduate Studies	4	6.7
**Experience in Critical Care Unit (years)**		
− < 1	16	26.7
− 1 < 5	38	**63.3**
− 5 < 10	3	5
− ≥ 10	3	5
***Mean ± SD***	4.56 ± 2.71	
**Training about Braden Scale**		
− Yes	9	15
− No	51	**85**

**Fig. 1:**
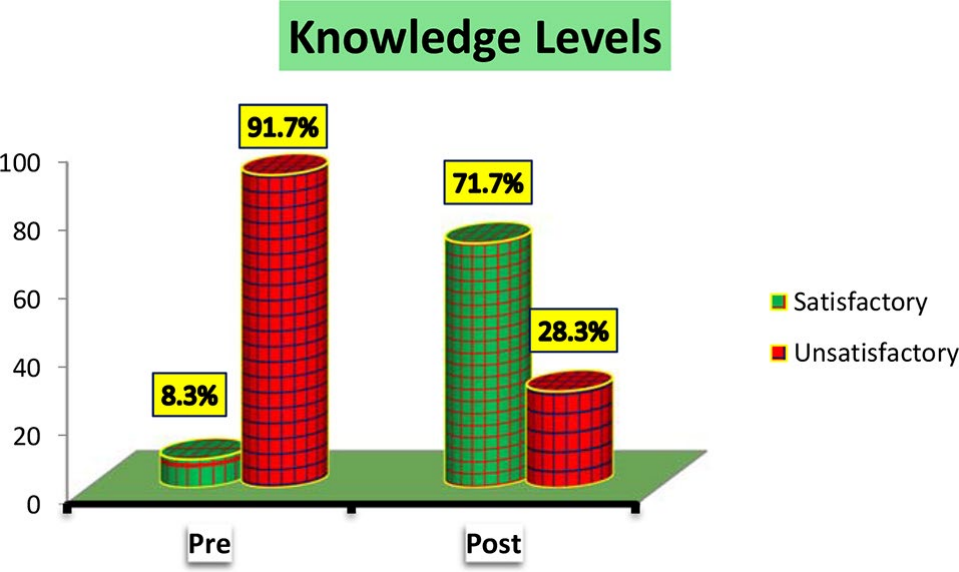
Studied nurses overall knowledge levels

Figure 1 shows that the majority of studied nurses (71.7%) had satisfactory knowledge levels after self-learning package implementation while the majority of them (91.7%) had unsatisfactory knowledge levels before self-learning package implementation.

**Fig. 2:**
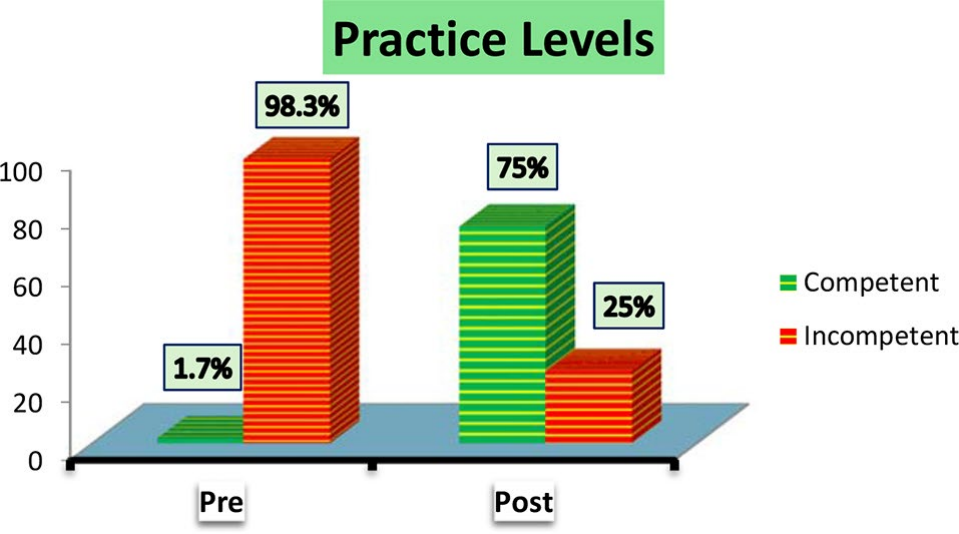
Studied nurses overall practice levels

Figure 2 illustrated that the majority of studied nurses (75%) had competent practice levels post self-learning package implementation while the majority of them (98.3%) had incompetent practice levels pre self-learning package implementation.

**Fig. 3:**
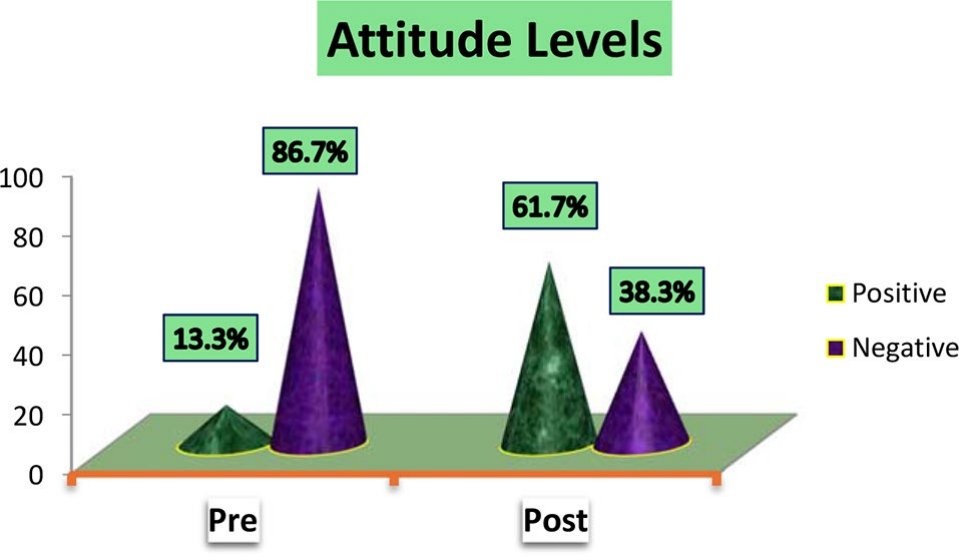
Studied nurses overall attitude levels

As shown in figure (3) the majority of studied nurses (61.7%) had Positive attitude levels after self-learning package implementation while the majority of them (86.7%) had negative attitude levels before self-learning package implementation.

Table 7 summarized the overall mean score of studied nurses’ knowledge, practice and attitude regarding use of Braden scale for predicting pressure ulcers pre and post self-learning package. Regarding studied nurses’ knowledge, the overall mean score of studied nurses’ knowledge regarding use of Braden scale after self-learning package implementation (21.61 ± 1.83) was higher as compared to pre self-learning package (7.38 ± 2.48). Using Paired sample t test revealed that this improvement in studied nurses’ knowledge after self-learning package implementation was statistically significant (t=-33.046, *P* = 0.000).

**Table 7:** Overall mean score of studied nurses’ knowledge, practice and attitude regarding use of Braden scale for predicting pressure ulcers pre and post self-learning package implementation (*n* = 60)

Study variables	Min - Max	Pre self-learning package	Post self-learning package	t-value	*P*-value
		Mean ± SD	Mean ± SD		
Anatomy	0–5	1.40 ± 1.07	4.36 ± 0.93	-17.355	0.000**
Pressure Ulcer	0–10	3.51 ± 1.65	8.48 ± 1.15	-18.456	0.000**
Braden Scale	0–10	2.46 ± 1.44	8.76 ± 1.09	-25.708	0.000**
**Overall Knowledge**	0–25	7.38 ± 2.48	21.61 ± 1.83	-33.046	0.000**
Sensation	0–8	3.43 ± 1.75	6.61 ± 1.43	-10.572	0.000**
Mobility	0–8	3.64 ± 1.93	7.01 ± 1.28	-10.968	0.000**
Activity	0–8	3.53 ± 1.74	7.05 ± 1.25	-12.021	0.000**
Moisture	0–8	1.28 ± 0.88	3.31 ± 0.85	-12.102	0.000**
Friction	0–8	2.53 ± 1.43	7.01 ± 1.39	-12.038	0.000**
Nutrition	0–8	3.53 ± 1.86	6.93 ± 1.41	-7.825	0.000**
**Overall Practice**	0–48	19.30 ± 6.65	41.11 ± 4.08	-19.890	0.000**
**Overall Attitude**	0–20	7.56 ± 2.80	16.15 ± 2.20	-19.239	0.000**

*t*: Paired sample *t* test, ** Highly significant at *P* < 0.01

Concerning studied nurses’ practice, the overall mean score of studied nurses’ practice regarding use of Braden scale after self-learning package implementation (41.11 ± 4.08) was higher as compared to pre self-learning package (19.30 ± 6.65). Using Paired sample t test revealed that this improvement in studied nurses’ practice after self-learning package implementation was statistically significant (t=-19.890, *P* = 0.000).

Concerning studied nurses’ Attitude, the overall mean score of studied nurses’ practice regarding use of Braden scale after self-learning package implementation (16.15 ± 2.20) was higher as compared to pre self-learning package (7.56 ± 2.80). Using Paired sample t test revealed that this improvement in studied nurses’ Attitude after self-learning package implementation was statistically significant (t=-19.239, *P* = 0.000).

Table 8 displayed the Correlation between studied nurses’ knowledge, practice and attitude regarding use of Braden scale for predicting pressure ulcers among critically ill patients pre and post self-learning package. Before self-learning package implementation, there were significant statistical correlation between studied nurses’ knowledge and their practice (*r* = 0.497, *P* = 0.000), between knowledge and attitude (*r* = 0.379, *P* = 0.001). Also, there were significant statistical correlation between studied nurses’ attitude and their practice (*r* = 0.438, *P* = 0.000). Also, after self-learning package implementation, there were significant statistical correlation between studied nurses’ knowledge and their practice (*r* = 0.467, *P* = 0.000), between knowledge and attitude (*r* = 0.407, *P* = 0.001). Also, there were significant statistical correlation between studied nurses’ attitude and their practice (*r* = 0.390, *P* = 0.000).

**Table 8:** Correlation between studied nurses’ knowledge, practice and attitude regarding use of Braden scale for predicting pressure ulcers pre and post self-learning package implementation (*n* = 60)

Phase	Study variables		Overall practice	Overall attitude
Pre self learning package	Overall Knowledge	*r*	0.497	0.379
		P-Value	0.000**	0.001**
	Overall Practice	*r*		0.438
		P-Value		0.000**
Post self-learning package	Overall Knowledge	*r*	0.467	0.407
		P-Value	0.000**	0.000**
	Overall Practice	*r*		0.390
		P-Value		0.000**

## Discussion

Regarding studied nurses’ demographic characteristics, the current study revealed that, more than half of the studied nurses aged from 18 to < 30 years old, with mean age 32.45 ± 6.78. This may be reflected in most of those nurses were newly graduated and of young age to tolerate the demanding nature of the work in the critical care unit.

This finding is in agreement with [22] who conduct a study and who reported that more than half of studied nurses were at the age group between 25-<35 years old with mean ± SD 33.1 ± 2.31.

Concerning the integration of the Braden Scale into nursing education varies across regions. In some countries, the scale is included in undergraduate nursing curricula, particularly in courses focused on risk assessment and patient care planning [15]. However, in Egypt, most nurses in this study reported limited exposure to formal training on the Braden Scale during their education. This finding aligns with research by [10], which highlighted gaps in training on pressure ulcer prevention among Egyptian nurses, often necessitating post-graduation learning through on-the-job training, additional courses, or self-education. Addressing these gaps by incorporating evidence-based tools like the Braden Scale into nursing school curricula can ensure graduates are better equipped for clinical practice and enhance the standard of patient care [23].

In relation to previous training courses about Braden scale, the current study revealed that the majority of the studied nurses didn’t attend any training courses about Braden scale. This finding may be due to the shortage of nursing staff and work overload which is considered a barrier for nurses to have time to attend training courses as well as lack of in-service training. This result is in agreement with [24] whose study title was ‘A Study to Assess the Effectiveness of Structured Teaching Program regarding Use of Braden Scale in Prevention of Pressure Sores in Terms of Knowledge among Staff Nurses at Selected Hospital, District Hisar (Haryana)’’ and found that, more than half of the studied nurses hadn’t attended any formal training about Braden scale. Meanwhile, this finding is contradicted with [5] whose study was entitled ‘Attitude of Nurses towards Use of Braden Scale’ and mentioned that, more than 60% of staff nurses attended class on use of Braden scale.

Concerning total nurses’ knowledge regarding use of Braden scale for predicting pressure ulcers among critically ill patients Fig. 1 pre and post implementation of self-learning package, the current study revealed that, majority of the studied nurses had unsatisfactory level of knowledge pre self-learning package implementation, while post implementation of self-learning package, more than two thirds of them had satisfactory level of knowledge about use of Braden scale for predicting pressure ulcers.

This improvement may be attributed to the present self-learning package using information, adequate sessions and practical content which was given to the studied nurses with the continuous explanations, reinforcement and feedback as well as sufficient materials provided for training. From the researchers’ point of view, the potential cause of unsatisfactory level of knowledge pre self-learning package implementation, that the majority of the studied nurses didn’t attend any in-service training program regarding use of Braden scale for predicting pressure ulcers. Also, more than one fifth of the studied nurses were diploma graduates, and their knowledge was obtained during school study years and it might be forgotten. Therefore, comprehensive educational sessions regarding Braden scale should be held regularly.

This finding goes in the same line with a study conducted by [25] titled “ Effectiveness of Structured Teaching Program on Knowledge and Attitude Regarding use of Braden Scale Among Staff Nurses Working in Different Hospitals of Moradabad ” who reported that about two thirds (61.2%) of staff nurses had inadequate knowledge about use of Braden scale in the pre-test, whereas in the post-test, the majority (82.5%) of them had adequate knowledge.

Moreover, this result agreed with [26] in a study titled “ A Study to Evaluate the Effectiveness of Structured Teaching Program on Knowledge and Practice regarding Braden Scale for Assessing Pressure Ulcer among Staff Nurses of Selected Hospitals ” and stated that minority of the studied nurses had good knowledge before the intervention of structured teaching program, whereas after structured teaching program, more than two thirds (73.33%) of the staff nurses had good knowledge regarding Braden scale for assessing pressure ulcer.

As regards to the total nurses’ practice pre and post implementation of self-learning package Fig. 2, the current study showed that the total mean score of all practices were improved post self-learning package implementation than pre implementation with highly statistically significant differences between all practices at pre and post implementation. This result may be attributed to the effectiveness of the practical content and comprehensive knowledge provided about the Braden scale for predicting pressure ulcers among critically ill patients.

This finding is supported by [27] who studied the “Effectiveness of PtP regarding Use of Braden Scale for Pressure Sore on Knowledge and Practices among Staff Nurses Working in selected Hospitals” and they reported that there was highly statistically significant difference in the mean of pre-and post-test practices score. Also [23], stated that there was a statistically significant difference between area wise pre- test and post- test practice scores regarding use of Braden scale for predicting pressure sore risk for bed fast patients.

In relation to nurses’ attitude pre and post implementation of self-learning package Fig. 3, the current study revealed that nurses’ attitude changed from negative to positive post self-learning package implementation with highly statistically significant differences between total mean score of attitude at pre and post implementation. This result could be attributed to many reasons such as improved knowledge and enhanced practice regarding the use of Braden scale for predicting pressure ulcers among critically ill patients, which is the better way to change the attitude of health professionals.

This result is consistent with [25] who mentioned that all participants in pre-test belonged to an unfavorable attitude and in the post-test, all of them belonged to a favorable attitude with statistically significant differences between pre and post test scores. Furthermore, this finding disagreed with [5], who assessed the attitude of nurses towards use of Braden scale and mentioned that majority of nurses had a favorable attitude and about one third had an unfavorable attitude regarding use of Braden scale for prevention of bed sore.

In addition, the present study revealed that there was a highly significant positive correlation between studied nurses’ knowledge and their practice pre and post self-learning package implementation. This result indicated that practice can be improved especially if linked with relevant scientific base of knowledge.

This result in the same line with [28] in a study titled “Effect of Implementing Standardized Preventive Guidelines for Pressure Ulcer on Nurses’ Performance " and said that there was positive relation between nurses’ practice with knowledge and attitude pre and post implementing standardized guidelines regarding the care of high-risk patients for pressure ulcers. On the other hand, this result is contradicted with [3] who stated that there was no statistically significant between the nurses’ knowledge and their practice.

This study demonstrated significant improvements in nurses’ knowledge, practices, and attitudes regarding the use of the Braden Scale for pressure ulcer prevention following the implementation of a self-learning package. These results align with findings by [29], who reported substantial gains in nurses’ knowledge after an educational intervention targeting pressure ulcer prevention. However, the magnitude of improvement in our study was higher, potentially due to the structured and flexible nature of the self-learning package, which allowed participants to learn at their convenience and engage with materials multiple times.

The improvement in practice scores observed in this study exceeds those reported by [7], who utilized traditional in-service training methods. One possible explanation for this discrepancy is the interactive and learner-driven design of the self-learning package, which may foster deeper understanding and application of theoretical knowledge in clinical settings. Furthermore, the cultural and organizational context of our study—conducted in an Egyptian ICU with limited prior training opportunities—may have amplified the intervention’s impact, as participants were more likely to benefit from structured educational support.

In terms of attitude shifts, this study observed a more pronounced positive change compared to findings by [10], While Soliman et al. noted some improvement in attitudes, the gains were modest, potentially due to differences in sample characteristics and intervention content. In our study, the inclusion of practical demonstrations and emphasis on clinical relevance may have contributed to a more substantial attitude change. Additionally, cultural factors, such as the high workload and limited resources typical of Egyptian ICUs, may have initially influenced negative attitudes, which the intervention successfully addressed.

The unique findings of this study emphasize the importance of self-directed learning in resource-limited settings. Unlike structured in-service training programs reported in previous studies [6], the self-learning package demonstrated scalability and feasibility, making it a viable solution for addressing gaps in nursing education, particularly in low- and middle-income countries like Egypt. Future studies should explore the long-term retention of knowledge, practice, and attitude improvements, as well as the potential for scaling self-learning packages across diverse healthcare settings.

A notable limitation of this study is the lack of assessment of long-term retention of improvements in nurses’ knowledge, practices, and attitudes following the self-learning package implementation. To address this, future studies should include follow-up evaluations at three, six-, or twelve-months post-intervention to determine the sustainability of the observed changes and their impact on clinical outcomes.

## Conclusion

This study explored the effect of a self-learning package on nurses’ performance related to the use of the Braden Scale for predicting pressure ulcers in critically ill patients. The findings revealed a marked improvement in nurses’ knowledge, practice, and attitude after the implementation of the educational package. Pre self learning packages most of studied nurses demonstrated low levels of knowledge, poor practice, and negative attitudes toward pressure ulcer risk assessment. However, after participating in the self-learning intervention, the majority exhibited clear gains in understanding, practical competence, and positive perception.

The results also showed significant correlations between knowledge, practice, and attitude both before and after the intervention, indicating that enhanced understanding contributed positively to improved clinical behavior and mindset. These findings emphasize the value of targeted educational strategies in empowering nurses with essential tools and competencies required for high-quality care in intensive care settings.

In the context of Egypt, the findings underscore critical gaps in knowledge and training regarding pressure ulcer prevention, as most nurses in this study reported no prior formal training on the Braden Scale. The high workload and limited availability of in-service training programs for nurses exacerbate this issue. These findings emphasize the need for tailored educational strategies that align with the specific challenges faced by Egyptian ICU nurses, including limited resources and time constraints.

Hospital administrators and policymakers in Egypt should prioritize the integration of evidence-based educational tools, such as self-learning packages, into continuous professional development programs. This approach can enhance the quality of care, reduce the incidence of pressure ulcers, and ultimately improve patient outcomes in critical care settings. Future research should replicate this study across other Egyptian healthcare facilities to further validate these findings and explore the scalability of self-learning interventions.

## Recommendations

The findings of this study have practical implications in nursing practice, education, research, and management. In practice, self-learning packages can empower nurses to improve their competency in pressure ulcer prevention, leading to enhanced patient outcomes. In education, incorporating self-directed learning into curricula can foster independent learning and skill development among nursing students. For research, the results highlight the need for further studies to explore the long-term impact of self-learning tools on nursing performance and patient care. In management, self-learning packages offer a cost-effective strategy for continuous professional development, addressing training gaps while optimizing resources. These applications underscore the potential of self-learning tools as a scalable and flexible solution to advance nursing competencies in diverse healthcare settings.

Self-learning package regarding the use of Braden scale for predicting pressure ulcers among critically ill patients should be available and applied in all intensive care units to be followed by all nurses and should be updated periodically. Replicated the study on large sample and in different geographical settings in order to generalize the results.

## Electronic supplementary material

Below is the link to the electronic supplementary material.

Supplementary Material 1

Supplementary Material 2

## Data Availability

The datasets generated during the current study are available in the [Mohamed H. Kotp] repository, The datasets during the current study available from the corresponding author on reasonable request. All data generated or analysed during this study are included in this published article.
