# Supplementary material for: Nurses’ performance regarding use of Braden scale for predicting pressure ulcers among critically ill patients: self learning package
Source: BMC Nurs. 2025 Jul 18;24:940. doi: 10.1186/s12912-025-03511-0 (PMC12273477; doi:10.1186/s12912-025-03511-0)
Supplement: Supplementary file 1 — Supplementary Material 1 [file 12912_2025_3511_MOESM1_ESM.pdf]

Instructions on Bedsores and Their Prevention Methods: A Self Learning Package

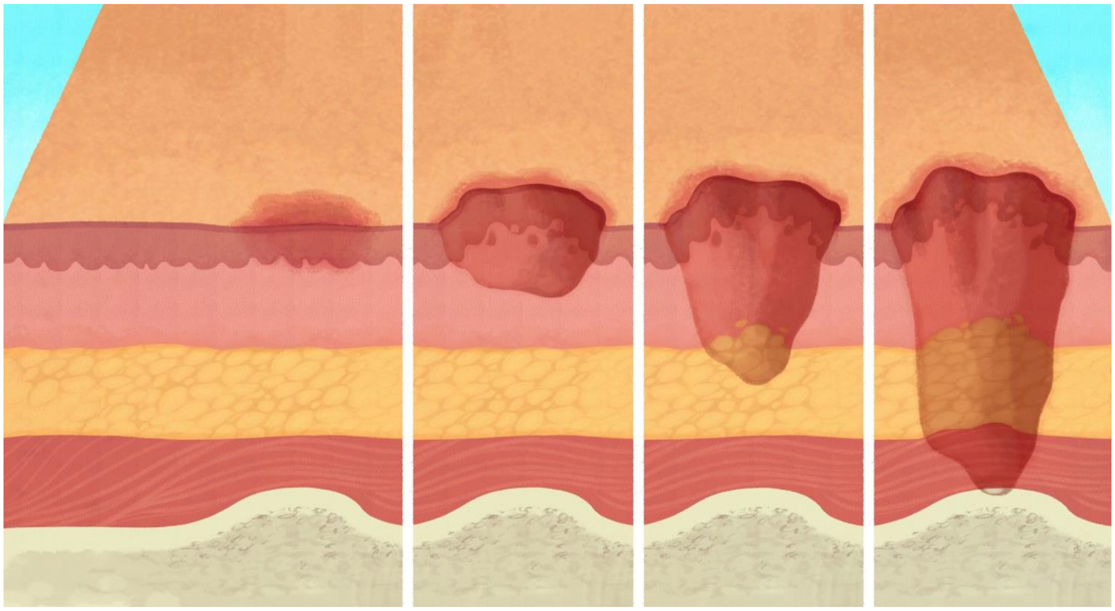

2024

Introduction

The human skin protects the body's organs and tissues from the effects of external factors such as germs, bacteria, harmful chemicals, heat, cold, and fluid penetration inside the body. The skin is the protective covering of the human body, and any cut or scratch allows germs and bacteria to enter and damage internal body tissues.Capillaries and blood vessels transport nutrition and oxygen to skin cells, as well as immune lymphatic cells to protect the skin from diseases and infections.

Skin cells die if they are subjected to pressure for more than two or three hours, especially when lying down, particularly under bony prominences, because blood does not reach them. Therefore, our bodies are programmed to move and turn during sleep, which does not happen in cases of coma, some aging diseases, and paralysis of the lower limbs, leading to bedsores caused by the death and shedding of skin cells, leaving the way open for harmful bodies to attack the sleeping patient's body.
This topic addresses the problem of bedsores in detail, explaining the factors and causes leading to this condition, the symptoms that appear, and ways to prevent and treat it.

Contents

- Definition of the skin
- Layers of the skin
- Functions of the skin
- Definition of bedsores
- Causes of bedsores
- People most at risk of bedsores
- Symptoms of bedsores
- Stages of bedsores
- Treatment methods of bedsores
- Prevention of bedsores
- Complications of bedsores
- Braden scale for predicting risk of bedsores

Definition of the skin:

The skin is the largest organ of the human body, and along with nails, hair, glands, and nerves, it forms the integumentary system that protects the body's internal organs from external influences and factors. The skin accounts for about 16% of the weight in adults, and its thickness varies depending on the location; for example, the skin covering the eyelids is thin, while the skin on the palms and soles is thick.

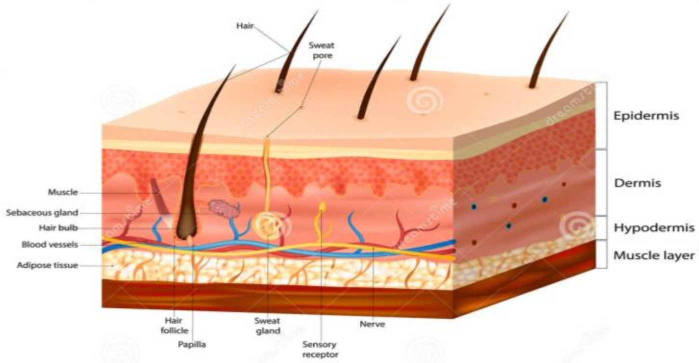

Layers of the skin:

The skin consists of three main layers:

1- Epidermis

It is the outer layer of the skin and is constantly renewing itself. The main functions of the epidermis include:

- Formation of new skin cells: New skin cells form at the base of the epidermis, replacing old cells that continuously shed.

- Giving the skin its color: Epidermal cells contain melanocytes that produce melanin, the pigment responsible for skin color.

- Protecting the skin: As the outer layer, the epidermis protects the skin from the external environment due to the keratin it contains, which gives strength and prevents dryness.

2- Dermis

It is the middle layer and the thickest of the skin layers. The dermis contains sweat glands, sebaceous glands, hair follicles, nerves, and blood vessels.

The dermis consists of collagen, a protein that gives skin its elasticity and strength.

Important functions of the dermis include:

Sensation (pain and touch): The dermis contains nerve endings that transmit sensations such as texture, pressure, pain, itchiness, and temperature differences to the brain.

Secretion of sweat and oils: Sweat glands help lower body temperature, and sebaceous glands secrete oils that moisturize and keep the skin soft.

Hair growth: Hair follicles in the dermis grow hair on the head, face, and body, helping regulate body temperature and protecting the body from injuries.

Fighting infection: The dermis contains lymphatic vessels, part of the lymphatic system, which help fight infections, germs, and protect the body from foreign substances.

3- Hypodermis (subcutaneous layer)

The deepest skin layer, mainly composed of fat, protects the body from extreme heat or cold.

This layer is a good energy reservoir as the body stores fat here. The fat also cushions internal organs such as muscles and bones.

Functions of the skin:

Key functions of the skin include:

Protection:

The skin protects the body from various factors such as physical or thermal injuries, harmful substances, moisture loss, and ultraviolet rays.

Body temperature regulation:

Skin helps maintain internal body temperature despite external temperature changes by responding through blood vessel dilation or constriction.

Sensation:

The skin contains nerve endings that transmit sensations of texture, pain, pressure, and heat to the brain.

Bedsores:

About 2.5 million people worldwide develop bedsores annually, especially the elderly, bedridden patients, and those with mobility disabilities requiring prolonged immobility. Bedsores can lead to serious complications if not treated early.

Definition of bedsores:

Bedsores are ulcers or injuries occurring on skin areas exposed to pressure due to lying in bed, sitting in a wheelchair, or wearing a cast for extended periods. Also known as pressure ulcers or decubitus ulcers, they occur when an immobile person remains in one position for long periods. Commonly affected bony areas include elbows, knees, lower back, buttocks, back of the head, and ankles.

Causes of bedsores:

Prolonged pressure on specific skin areas is the main cause, leading to interrupted blood supply, causing cell death. Other contributing factors include:

- Moisture
- Poor circulation
- Malnutrition
- Diabetes
- Thin skin
- Poor hygiene and repeated contamination with urine or feces (especially in the elderly or those wearing diapers)
- Friction (skin becomes thinner and damaged due to rubbing or sheet pulling)
- Long-term cast wearing

People most at risk:

Anyone can develop bedsores, but risks increase for immobile individuals. Factors include:

Aging (skin becomes thinner, fragile, and dry) Urinary or fecal incontinence, increasing skin damage risk Obesity, increasing skin pressure when lying down Medical conditions affecting blood supply or skin fragility (diabetes, peripheral artery disease, kidney failure, heart failure, multiple sclerosis, Parkinson's) Poor nutrition, impacting skin condition.

Symptoms of bedsores:

- Bedsores usually start as a painful red area, progressively worsening. Symptoms include:
- Skin color changes (red, purple, blue, or darkened skin) Texture changes (hard, warm, spongy, painful, or itchy area)
- Open wounds with possible fluid or pus, potentially deep tissue damage
- Infection signs (increased pain, pus, discoloration, fever, heat around sore)

Stages of bedsores:

Bedsores develop in four stages:

- **Stage 1:**
  Red or purple warm swollen area, with pain and itching.
- **Stage 2:**
  More damage, open wounds or superficial skin erosion, severe pain, skin discoloration.
- **Stage 3:**
  Deep crater from tissue and fat damage.
- **Stage 4:**

Severe damage involving muscles, tendons, possible bone exposure, infection with pus and extensive tissue damage.

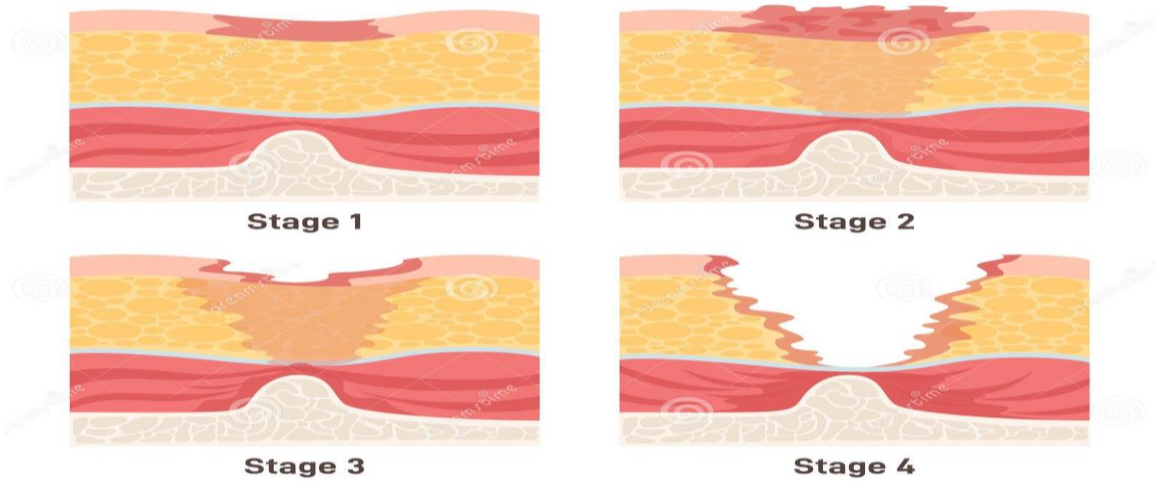

Treatment methods:

- Treatment depends on severity and patient health, possibly taking days to years. May involve surgery. General methods:
- Regular repositioning to improve blood flow and skin ventilation
- Use of air mattresses
- Healthy diet and hydration
- Cleaning wounds and dressing changes
- Use of topical and oral antibiotics
- Protective creams like petroleum jelly
- Surgery to remove dead tissue and skin grafts for severe cases
- Advanced treatments like hyperbaric oxygen therapy

Prevention:

- Regular skin inspection, especially for immobile patients
- Repositioning every two hours
- Soft cushions to reduce pressure
- Skin care to keep dry and clean
- Balanced nutrition rich in vitamins and proteins
- Avoid smoking
- Skin care tips and body position adjustments provided for patients.

Complications:

- Untreated sores can lead to life-threatening infections like sepsis and cellulitis, bone and joint infections, and organ failure.
- Braden scale for risk prediction:
- Developed in 1987 to assess risk of bedsores by evaluating six criteria: sensory perception, moisture, activity, mobility, nutrition, friction/shear. Scoring helps healthcare providers determine risk level and preventive measures.

Braden scale for pressure ulcer

Here is a **translated, enhanced, and detailed English version** of your provided Arabic content about the **Braden Scale for Predicting Pressure Ulcer Risk** — suitable for inclusion in your manuscript, training materials, or supplementary file:

Braden Scale for Predicting Pressure Ulcer Risk

The **Braden Scale** is a widely used clinical assessment tool developed in 1987 by **Barbara Braden** and **Nancy Bergstrom**. Its primary purpose is to help healthcare professionals—especially nurses—systematically assess a patient's risk of developing **pressure ulcers** (also known as pressure injuries or bedsores).

Assessment Dimensions of the Braden Scale

The scale evaluates six key dimensions that are critical in pressure ulcer development:

1. **Sensory Perception**

   This dimension assesses the patient's ability to detect and respond meaningfully to discomfort related to pressure on parts of the body. It includes evaluating the patient's level of consciousness and responsiveness to stimuli such as pain or discomfort, which can influence their ability to shift position or request assistance.

2. **Moisture**
   Continuous exposure to moisture can cause the skin to become macerated, increasing the risk of skin breakdown. This category evaluates how often the skin is exposed to moisture from sources such as perspiration, wound drainage, or incontinence.

3. **Activity**
   This item measures the patient's physical activity level. Reduced mobility, especially prolonged bed rest or use of a wheelchair, can accelerate muscle atrophy and tissue ischemia, increasing the risk of ulcer formation.

4. **Mobility**
   This assesses the patient's ability to change and control body position independently. Patients with limited mobility are more prone to prolonged pressure on specific body areas, contributing to ulcer development.

5. **Nutrition**
   This category evaluates the patient's usual food intake patterns. Poor nutrition—such as inadequate protein or caloric intake—impairs wound healing and increases skin vulnerability to breakdown.

6. **Friction and Shear**

   This dimension examines the degree of assistance the patient requires to move and whether they experience sliding in bed or a chair. Shear occurs when the skin remains in place while underlying tissues shift, leading to capillary damage and tissue breakdown.

Scoring Method

Each of the six dimensions is scored as follows:

- Most subscales (Sensory Perception, Moisture, Activity, Mobility, Nutrition) are rated from **1 to 4**, where:
  - **1 = severely limited**
  - **4 = no impairment**
- The **Friction and Shear** subscale is rated from **1 to 3**.

The total score ranges from **6 to 23**:

- **Lower scores** indicate **higher risk** of developing pressure ulcers.
- **Higher scores** indicate **lower risk**.

Risk Stratification Based on Total Score

Total Braden Score	Risk Level
**≤ 9**	Very High Risk
**10–12**	High Risk
**13–14**	Moderate Risk
**15–18**	Mild Risk
**19–23**	No Apparent Risk

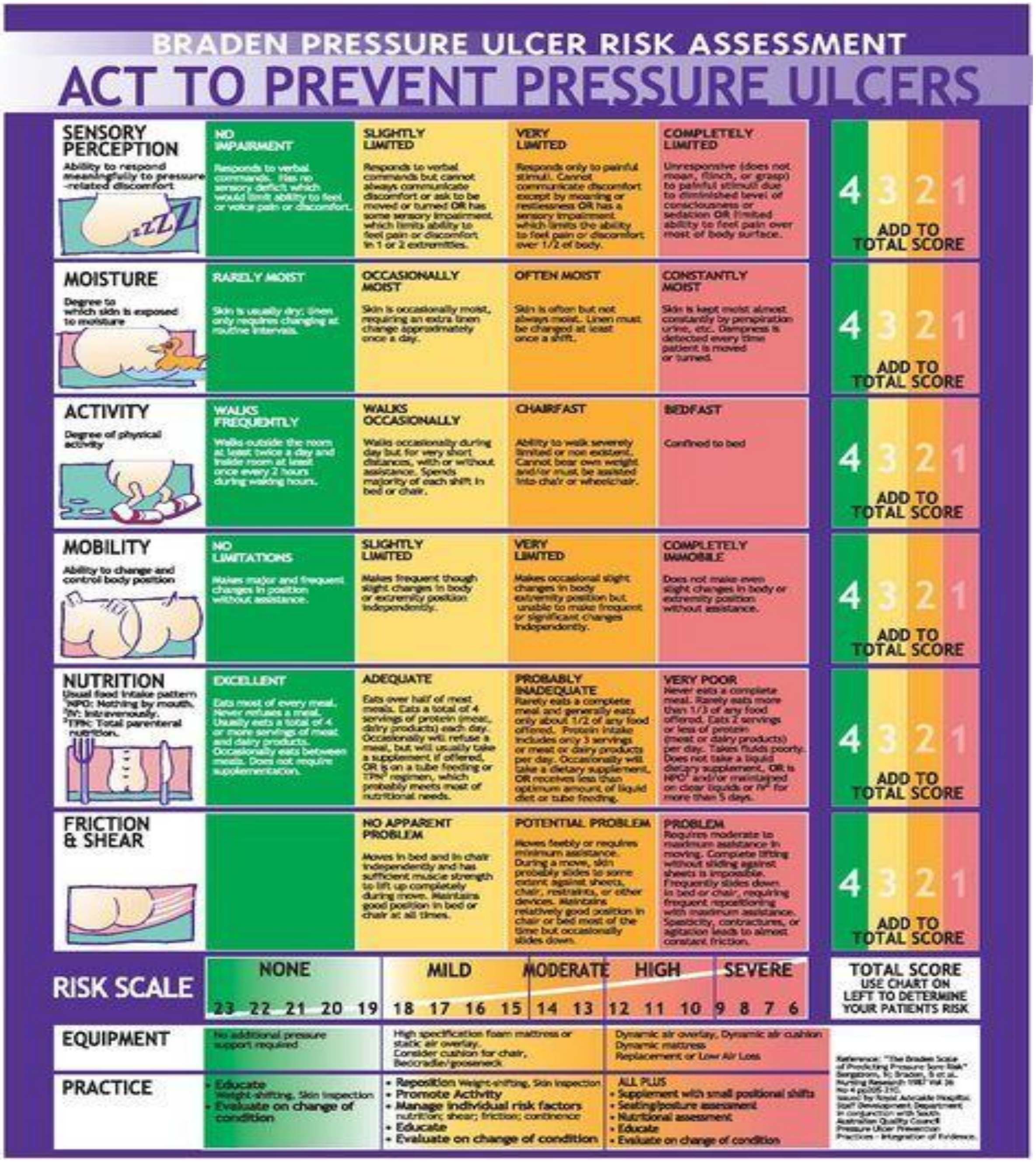

BRADEN PRESSURE ULCER RISK ASSESSMENT

ACT TO PREVENT PRESSURE ULCERS

**SENSORY PERCEPTION** Ability to respond meaningfully to pressure-related discomfort	**NO IMPAIRMENT** Responds to verbal commands. Has no sensory deficit which would limit ability to feel or voice pain or discomfort.	**SLIGHTLY LIMITED** Responds to verbal commands but cannot always communicate discomfort or ask to be moved or turned OR has some sensory impairment which limits ability to feel pain or discomfort in 1 or 2 extremities.	**VERY LIMITED** Responds only to painful stimuli. Cannot communicate discomfort except by moaning or restlessness OR has a sensory impairment which limits the ability to feel pain or discomfort over 1/2 of body.	**COMPLETELY LIMITED** Unresponsive (does not moan, flinch, or grasp) to painful stimuli due to diminished level of consciousness or sedation OR limited ability to feel pain over most of body surface.	4 3 2 1 ADD TO TOTAL SCORE
**MOISTURE** Degree to which skin is exposed to moisture	**RARELY MOIST** Skin is usually dry; linen only requires changing at routine intervals.	**OCCASIONALLY MOIST** Skin is occasionally moist, requiring an extra linen change approximately once a day.	**OFTEN MOIST** Skin is often but not always moist. Linen must be changed at least once a shift.	**CONSTANTLY MOIST** Skin is kept moist almost constantly by perspiration urine, etc. Dampness is detected every time patient is moved or turned.	4 3 2 1 ADD TO TOTAL SCORE
**ACTIVITY** Degree of physical activity	**WALKS FREQUENTLY** Walks outside the room at least twice a day and inside room at least once every 2 hours during waking hours.	**WALKS OCCASIONALLY** Walks occasionally during day but for very short distances, with or without assistance. Spends majority of each shift in bed or chair.	**CHAIRFAST** Ability to walk severely limited or non existent. Cannot bear own weight and/or must be assisted into chair or wheelchair.	**BEDFAST** Confined to bed	4 3 2 1 ADD TO TOTAL SCORE
**MOBILITY** Ability to change and control body position	**NO LIMITATIONS** Makes major and frequent changes in position without assistance.	**SLIGHTLY LIMITED** Makes frequent though slight changes in body or extremity position independently.	**VERY LIMITED** Makes occasional slight changes in body extremity position but unable to make frequent or significant changes independently.	**COMPLETELY IMMOBILE** Does not make even slight changes in body or extremity position without assistance.	4 3 2 1 ADD TO TOTAL SCORE
**NUTRITION** Usual food intake pattern ¹NPO: Nothing by mouth. ²IV: Intravenously. ³TPN: Total parenteral nutrition.	**EXCELLENT** Eats most of every meal. Never refuses a meal. Usually eats a total of 4 or more servings of meat and dairy products. Occasionally eats between meals. Does not require supplementation.	**ADEQUATE** Eats over half of most meals. Eats a total of 4 servings of protein (meat, dairy products) each day. Occasionally will refuse a meal, but will usually take a supplement if offered. OR is on a tube feeding or TPN³ regimen, which probably meets most of nutritional needs.	**PROBABLY INADEQUATE** Rarely eats a complete meal and generally eats only about 1/2 of any food offered. Protein intake includes only 3 servings or meat or dairy products per day. Occasionally will take a dietary supplement, OR receives less than optimum amount of liquid diet or tube feeding.	**VERY POOR** Never eats a complete meal. Rarely eats more than 1/3 of any food offered. Eats 2 servings or less of protein (meat or dairy products) per day. Takes fluids poorly. Does not take a liquid dietary supplement, OR is NPO¹ and/or maintained on clear liquids or IV² for more than 5 days.	4 3 2 1 ADD TO TOTAL SCORE
**FRICTION & SHEAR**		**NO APPARENT PROBLEM** Moves in bed and in chair independently and has sufficient muscle strength to lift up completely during move. Maintains good position in bed or chair at all times.	**POTENTIAL PROBLEM** Moves feebly or requires minimum assistance. During a move, skin probably slides to some extent against sheets, chair, restraints, or other devices. Maintains relatively good position in chair or bed most of the time but occasionally slides down.	**PROBLEM** Requires moderate to maximum assistance in moving. Complete lifting without sliding against sheets is impossible. Frequently slides down in bed or chair, requiring frequent repositioning with maximum assistance. Spasticity, contractures, or agitation leads to almost constant friction.	4 3 2 1 ADD TO TOTAL SCORE

RISK SCALE	NONE	MILD	MODERATE	HIGH	SEVERE	TOTAL SCORE USE CHART ON LEFT TO DETERMINE YOUR PATIENTS RISK
	23 22 21 20 19	18 17 16 15	14 13	12 11 10	9 8 7 6	

**EQUIPMENT**	No additional pressure support required	High specification foam mattress or static air overlay. Consider cushion for chair, Bedcradle/gooseneck	Dynamic air overlay, Dynamic air cushion Dynamic mattress Replacement or Low Air Loss	
**PRACTICE**	• Educate Weight-shifting, Skin inspection • Evaluate on change of condition	• Reposition Weight-shifting, Skin inspection • Promote Activity • Manage individual risk factors nutrition; shear; friction; continence • Educate • Evaluate on change of condition	ALL PLUS • Supplement with small positional shifts • Seating/posture assessment • Nutritional assessment • Educate • Evaluate on change of condition	Reference: "The Braden Scale of Predicting Pressure Sore Risk" Bergstrom, N; Braden, B et al. Nursing Research 1987 Vol 36 No 4 pp205-210. Issued by Royal Adelaide Hospital Staff Development Department in conjunction with South Australian Quality Council Pressure Ulcer Prevention Practices - Integration of Evidence.

References:

- Berlowitz D. Clinical staging and management of pressure-induced skin and soft tissue injury. https://www.uptodate.com/contents/search. Accessed Nov. 14, 2023.
- Berlowitz D. Epidemiology, pathogenesis and risk assessment of pressure-induced skin and soft tissue injury. https://www.uptodate.com/contents/search. Accessed Nov. 14, 2023.
- Berlowitz D. Prevention of pressure-induced skin and soft tissue injury. https://www.uptodate.com/contents/search. Accessed Nov. 14, 2023.
- Briggs JK. Diaper (absorbant pad) rash. In: Triage Protocols for Aging Adults. Wolters Kluwer; 2019.
- Ferri FF. Pressure injury. In: Ferri's Clinical Advisor 2024. Elsevier; 2024. https://www.clinicalkey.com. Accessed Nov. 14, 2023.
- James WD, et al. Dermatoses resulting from physical factors. In: Andrews' Diseases of the Skin: Clinical Dermatology. 13th ed. Elsevier; 2020. https://www.clinicalkey.com. Accessed Nov. 14, 2023.
- Lebwohl MG, et al. Superficial and deep ulcers. In: Treatment of Skin Disease: Comprehensive Therapeutic Strategies. 6th ed. Elsevier; 2022. https://www.clinicalkey.com. Accessed Nov. 14, 2023.
- Neligan PC, et al., eds. Pressure sores. In: Plastic Surgery. 5th ed. Elsevier; 2024. https://www.clinicalkey.com. Accessed Nov. 14, 2023.
- Pressure injuries. Merck Manual Professional Version. https://www.merck.com/mmpe/sec10/ch126/ch126a.html. Accessed Nov. 14, 2023.
- Safe skin, safe patients: The value of patient hygiene-clinical consensus statement. AJN, American Journal of Nursing. 2023; doi:10.1097/01.NAJ.0000938764.42102.75.
- Sominidi Damodaran S (expert opinion). Mayo Clinic. Nov. 21, 202
- Tleyjeh IM, et al. Infectious complications of pressure-induced skin and soft tissue injury. https://www.uptodate.com/contents/search. Accessed Nov. 14, 2023.
- Townsend CM Jr, et al. Plastic surgery. In: Sabiston Textbook of Surgery: The Biological Basis of Modern Surgical Practice. 21st ed. Elsevier; 2022. https://www.clinicalkey.com. Accessed Nov. 14, 2023.
- Visconti AJ, et al. Pressure injuries: Prevention, evaluation and management. American Family Physician. 2023; https://www.clinicalkey.com. Accessed Nov. 14, 2023.
