# Supplementary material for: Nurses’ performance regarding use of Braden scale for predicting pressure ulcers among critically ill patients: self learning package
Source: BMC Nurs. 2025 Jul 18;24:940. doi: 10.1186/s12912-025-03511-0 (PMC12273477; doi:10.1186/s12912-025-03511-0)
Supplement: Supplementary file 2 — Supplementary Material 2 [file 12912_2025_3511_MOESM2_ESM.pdf]

Tool (I): Structured questionnaire

➢ **Code no:** ☐

Part-I: Nurses' demographic data:

Age (years):

18 - <30 ☐ 30 - <45 ☐ 40- ≤ 60 ☐

Gender:

Male ☐ Female ☐

Educational level:

Nursing diploma ☐ Technical institute of nursing ☐

Bachelor of Nursing ☐ Postgraduate studies ☐

Years of experience in critical care unit:

<1year ☐ 1-<5 years ☐

5-10years ☐ >10 years ☐

Previous training courses about Braden scale:

Yes ☐ No ☐

Part-II: Nurses' knowledge assessment questionnaire:

- **Skin anatomy:**

1. The skin is the body's largest and primary protective organ that is primarily made up of:

A. Two layers.
B. Three layers.
C. Four layers.
D. Five layers.

2. The outermost layer of skin that provides a waterproof barrier and contributes to skin tone is called:

A. Epidermis.
B. Hypodermis.
C. Intradermis.
D. Dermis.

3. The functions of the skin include the following except:

A. Protection against microorganisms.
B. Sensation of pain, temperature and touch.
C. Vitamin C production.
D. Regulation of body temperature.

4. The skin layer that found beneath the epidermis and contains hair follicles, blood vessels and sweat glands is called:

A. Dermis.
B. Hypodermis.
C. Intradermis.
D. Epidermis.

5. The deepest skin layer that made of fat and connective tissue is called:

A. Intradermis .
B. Hypodermis.
C. Dermis.
D. Epidermis.

- <u>**Pressure ulcer:**</u>

6. Pressure ulcer can be defined as localized injury to the skin and/or underlying tissue usually over a bony prominence that occurs as a result of:

A. Prolonged pressure.

B. Shear.

C. Excessive pressure in combination with shear and/or friction.

D. Adequate nutrition.

7. Pressure ulcer also known as:

A. Skin abrasion.

B. Skin loss.

C. Decubitus ulcers.

D. Sacral ulcers.

8. the most vulnerable areas for pressure ulcer are:

A. The sacrum, iliac crest, shoulders.

B. Inner or lateral aspect of the knees.

C. Prominences of the head, ankle, the heel and elbow.

D. All of the above.

9. The risk factors for the development of pressure ulcer include the following <u>except:</u>

A. Adequate level of mobility.

B. Old age (>65 years).

C. Pre-existing chronic illness such as urogenital disorders and stroke.

D. Altered level of consciousness.

10. Stage 1 pressure injury is called:

A. Partial-thickness skin loss.

B. Non-blanchable erythema.

C. Full-thickness skin loss.

D. Full-thickness skin and tissue loss.

11. Stage 2 pressure injury is characterized by:

A. Skin loss with exposed dermis and the wound bed is viable and moist.

B. Intact skin with localized area of erythema.

C. Skin loss with visible adipose tissues.

D. Skin and tissue loss with exposed muscle, tendon, or bone.

12. The key measures for preventing pressure ulcers in critically ill patients include the following <u>except:</u>

A. Avoiding use of support surfaces as air matters and cushions.

B. Providing adequate dietary intake of protein and calories.

C. Keeping patient ' skin clean and dry.

D. A turning schedule should be written and placed at the bed side.

13. Critically ill patients who confined to the bed should be repositioned at least every:

A. Two hours.

B. Four hours.

C. Five hours.

D. Six hours.

14. The early sign of pressure ulcer development include:

A. Open sore.

B. Intact or ruptured serum-filled blister.

C. Discolored patches of the skin that don’t change color when pressed.

D. Blood-filled blister.

15. The life-threatening complications of pressure ulcer include:

A. Septicemia.

B. Hypovolemic shock.

C. Prolonged hospital length of stay.

D. Impaired mobility.

- <u>**Braden scale:**</u>

16. Braden scale can be defined as clinically reliable and valid assessment tool that mainly developed to:

A. Predict patients at risk for developing a pressure ulcer.
B. Increase incidence of pressure ulcers in critically ill patients.
C. Improve quality of nursing care.
D. Provide comprehensive patient ' skin assessment.

17. Braden scale consists of …………… domains.

A. Five.
B. Six.
C. Seven.
D. Eight.

18. When to use the Braden scale to assess the patient:

A. Every shift.
B. Within 8 hours of patient 'admission.
C. On transition of care.
D. All of the above.

19. The Braden scale includes the following subscales <u>except:</u>

A. Sensory perception.
B. Activity level.
C. Sleeping pattern.
D. Nutritional status.

20. On the Braden scale interpretation, the lowest score refers to:

A. Mild risk.
B. Moderate risk
C. High risk
D. Severe risk.

21. Total score of Braden scale ranging from:

A. 6-21.

B. 6-22

C. 6-23

D. 6-24.

22. All Braden scale sections are scored from 1 to 4, except the friction and shear section, which scored from:

A. 1-3.

B. 1-2.

C. 1-4.

D. 1-5.

23. If your patient's Braden score is 16, what risk level does this patient fall into?

A. Mild risk.

B. Moderate risk

C. High risk

D. Severe risk.

24. If your patient's Braden score is 10, what risk level does this patient fall into?

A. Mild risk.

B. Moderate risk

C. High risk

D. Severe risk.

25. If your patient's Braden score is 13, what risk level does this patient fall into?

A. Mild risk.

B. Moderate risk.

C. High risk.

D. Severe risk.

Tool (II): Nurses' practice observational checklist

➢ **Code no:** ☐

Braden Risk Assessment Tool

Category	Description	Score	Correctly done	Incorrectly done	Not done
**Sensory Perception- Ability to respond meaningfully to pressure related discomfort**	**Completely Limited:** Unresponsive (does not moan, flinch or grasp) to painful stimuli due to diminished level of consciousness or sedation. **OR,** limited ability to feel pain over most of body surface.	**1**			
	**Very Limited:** Responds to only painful stimuli. Cannot communicate discomfort except by moaning or restlessness; **OR** has sensory impairment that limits the ability to feel pain or discomfort over half of body.	**2**			
	**Slightly Limited:** Responds to verbal commands, but cannot always communicate discomfort or need to be turned; **OR**, has sensory impairment that limits the ability to feel pain or discomfort in one or two extremities.	**3**			
	**No Impairment:** Responds to verbal commands. Has no sensory deficit that would limit ability to feel or communicate pain or discomfort.	**4**			
**Mobility- Ability to change and maintain own position**	**Completely immobile:** Does not make even slight changes in body or extremity position without assistance.	**1**			
	**Very limited:** Makes occasional slight changes in body or extremity position but unable to make frequent or significant changes independently.	**2**			
	**Slightly limited:** Makes frequent though slight changes in body or extremity position independently	**3**			
	**No limitations:** makes major and frequent changes in position without assistance.	**4**			

**Activity- Degree of physical activity**	**Bedfast:** confined to bed (can’t sit at all).	**1**			
	**Chairfast:** Ability to walk severely limited or non-existent. Cannot bear own weight and/or must be assisted into chair or wheelchair.	**2**			
	**Walks occasionally:** walks occasionally during day, but for very short distances, with or without assistance. Spends majority of each shift in bed or chair.	**3**			
	**Walks frequently:** Walks outside the room at least twice a day and inside room at least once every 2 hours during waking hours.	**4**			
**Moisture- Degree to which skin is exposed to moisture**	**Constantly moist:** skin is kept moist almost constantly by perspiration, urine, drainage etc. Dampness is detected every time patient is moved or turned.	**1**			
	**Very moist:** Skin is often, but not always, moist. Linen must be changed at least every 8 hours. Dry 2-3 hours at a time	**2**			
	**Occasionally moist:** Skin is occasionally moist, requiring linen change every 12 hours	**3**			
	**Rarely moist:** Skin is usually dry, linen only requires changing every 24 hours.	**4**			
**Friction and Shear**	**Problem:** Requires moderate to maximum assistance in moving. Complete lifting without sliding against sheets is impossible. Frequently slides down in bed or chair, requiring frequent repositioning with maximum assistance.	**1**			
	**Potential problem:** Moves feebly or requires minimum assistance. During a move, skin probably slides to some extent against sheets, chair, restraint or other devices. Maintains relative good position in chair or bed most of the time but occasionally slides down.	**2**			
	**No apparent problem:** Able to completely lift patient during a position change, moves in bed and in chair independently and has sufficient muscle strength to lift completely during move.	**3**			

**Nutrition**	**Very poor:** NPO and/or maintained on clear fluids, or IVs for more than 5 days OR never eats a complete meal. Rarely eats more than 1/3 of any food offered. Protein intake includes only 2 servings of meat or dairy products per day. Takes fluids poorly.	**1**			
	**Inadequate:** Is on a liquid diet or tube feedings/TPN, which provide inadequate calories and minerals for age OR rarely eats a complete meal and generally eats only half of any food offered. Protein intake includes only 3 servings of meat or dairy products per day.	**2**			
	**Adequate:** Is on tube feedings OR eats over half of most meals. Eats a total of 4 servings of protein each day. Occasionally eats between meals. Does not require supplementation.	**3**			
	**Excellent:** Is on TPN, which provides adequate calories and minerals for age OR Is on a normal diet providing adequate calories for age. For example, eats most of every meal. Never refuses a meal. Usually eats a total of 4 or more servings of meat and dairy products. Occasionally eats between meals. Does not require supplementation.	**4**			
**TOTAL SCORE**	▪ Mild risk- 15-18.<br>▪ Moderate risk - 13-14.<br>▪ High risk - 10-12.<br>▪ Severe risk - $\leq$9.				

Tool (III): Nurses' attitude questionnaire

➢ **Code no:** ☐

Please read the following sentences and check your opinion about it:

Items	Agree	Uncertain	Disagree
1. The use of Braden scale significantly reduce pressure ulcer in critically ill patients.			
2. Braden scale scoring should be regularly carried out on all patients during their stay in the critical care unit.			
3. Continuous assessment of patients using Braden scale is time consuming.			
4. The application of Braden scale will result in increased quality of nursing care.			
5. My clinical judgment is better than Braden scale scoring.			
6. All critically ill patients are at potential risk of developing pressure ulcers.			
7. Braden scale scoring is lower priority than other areas of care when providing care for critically ill patients.			
8. Braden scale helps health care providers to take specific measures to prevent pressure ulcers.			
9. It is necessary to interpret Braden scale correctly and providing suitable nursing interventions.			
10. Pressure ulcer treatment is of greater priority than pressure ulcer prevention.			

References

Abena, P.A. (2023). Study to Assess Knowledge of Braden Scale for Pressure Ulcer among ICU Staff Nurses. International Journal of Creative Research Thoughts (IJCRT); 11(8):936-959.

Edward, M. I., Ajibade, O. S., Adewoyin, F. R., & Adeoyin, A. B. (2021). Knowledge and Perception of Nurses on use of Braden Scale in Predicting Patients' Pressure Ulcer Risks in Selected Hospitals in Ondo State. Bayero Journal of Nursing and Health Care; 3(1): 748-757.

Leahy, D.H. (2023). The Impact Braden Scale Education on Nurses' Knowledge regarding Its Use, Doctorate thesis in Nursing practice, Faculty of the Graduate School of Arts and Sciences, Georgetown University. PP. 37-45.

Nazir, A. (2021). Assessment of Knowledge of Nurses about Prevention of Pressure Ulcer at Private Hospital in Lahore. Global Scientific Journal; 9(9):807-905.

Niyongabo, E.,Gasaba, E., Niyonsenga, P., Ndayizeye, M.,Ninezereza, J.B., Nsabimana, D., Nshimirimana, A. & Abakundanye, S. (2022). Nurses' Knowledge, Attitudes and Practice regarding Pressure Ulcers Prevention and Treatment. Open Journal of Nursing; 12 (5): 316-333.

Soliman, E. S., Ragheb, M. M., Abd El-Salam, H., & Mohamed, S.H. (2022). Effect of an Educational Program on Nurses' Performance Regarding Reducing Pressure Ulcer and Safety of Immobilized Patients. Benha Journal of Applied Sciences (BJAS); 3(2):856-871.

Suma, K., Sanjay, M., & Sharon, K. (2021). A Study to Evaluate the Effectiveness of Structured Teaching Programme (STP) on Knowledge Regarding Use of Braden Scale for Predicting Pressure Sore Risk Among

Student Nurses in KLEs Institute of Nursing Sciences, Hubballi. International Journal of Recent Innovations in Medicine and Clinical Research; 3(3):7-13.
